# Bioprinting Cell- and Spheroid-Laden Protein-Engineered Hydrogels as Tissue-on-Chip Platforms

**DOI:** 10.3389/fbioe.2020.00374

**Published:** 2020-04-28

**Authors:** Daniela F. Duarte Campos, Christopher D. Lindsay, Julien G. Roth, Bauer L. LeSavage, Alexis J. Seymour, Brad A. Krajina, Ricardo Ribeiro, Pedro F. Costa, Andreas Blaeser, Sarah C. Heilshorn

**Affiliations:** ^1^Department of Materials Science & Engineering, Stanford University, Stanford, CA, United States; ^2^Stanford Medical School, Institute for Stem Cell Biology and Regenerative Medicine, Stanford University, Stanford, CA, United States; ^3^Department of Bioengineering, Stanford University, Stanford, CA, United States; ^4^Biofabics, Porto, Portugal; ^5^Institute for BioMedical Printing Technology, Technical University of Darmstadt, Darmstadt, Germany

**Keywords:** protein engineered hydrogel, bioink, bioprinting, 3D cell culture, tissue model

## Abstract

Human tissues, both in health and disease, are exquisitely organized into complex three-dimensional architectures that inform tissue function. In biomedical research, specifically in drug discovery and personalized medicine, novel human-based three-dimensional (3D) models are needed to provide information with higher predictive value compared to state-of-the-art two-dimensional (2D) preclinical models. However, current *in vitro* models remain inadequate to recapitulate the complex and heterogenous architectures that underlie biology. Therefore, it would be beneficial to develop novel models that could capture both the 3D heterogeneity of tissue (e.g., through 3D bioprinting) and integrate vascularization that is necessary for tissue viability (e.g., through culture in tissue-on-chips). In this proof-of-concept study, we use elastin-like protein (ELP) engineered hydrogels as bioinks for constructing such tissue models, which can be directly dispensed onto endothelialized on-chip platforms. We show that this bioprinting process is compatible with both single cell suspensions of neural progenitor cells (NPCs) and spheroid aggregates of breast cancer cells. After bioprinting, both cell types remain viable in incubation for up to 14 days. These results demonstrate a first step toward combining ELP engineered hydrogels with 3D bioprinting technologies and on-chip platforms comprising vascular-like channels for establishing functional tissue models.

## Introduction

Three-dimensional (3D) cell culture systems that model the microenvironment of tissues and organs are expected to yield results with higher predictive value in drug discovery, preclinical testing, and personalized medicine (Langhans, 2018). It is well-accepted that 3D culture systems that mimic key factors of native extracellular matrix (ECM) are more representative of the *in vivo* microenvironment than comparative two-dimensional (2D) cultures (Petersen et al., 1992; Ravi et al., 2015). For example, 3D cancer models have shown more physiologically relevant outcomes in migration and invasion assays compared to 2D models (Katt et al., 2016). However, existing 3D models remain inadequate to recapitulate the complex and heterogenous architectures present *in vivo*. In particular, vascularization is typically absent from many 3D tissue models (Zhang et al., 2016). Vascular tissue interfaces are particularly important in *in vitro* models of the neural stem cell niche (Tavazoie et al., 2008), blood-brain-barrier (Brown et al., 2015), and *in vitro* models of cancer metastasis (Carey et al., 2013; Curtin et al., 2018).

Microfluidic and on-chip technologies are experimental models that can include dynamic vascular-like channels (Cochrane et al., 2019). In a recent study, a low permeability microfluidic platform was developed for screening pharmaceuticals that target neurodegenerative diseases (Bang et al., 2017). Although such platforms have shown vascular permeability comparable to reported *in vivo* studies, they fail to recapitulate the 3D architecture of the native tissue, as cells are cultured on 2D polydimethylsiloxane (PDMS) substrates. *In vitro* models of the neural stem cell niche commonly use random co-culture mixtures or transwell inserts that do not mimic the spatial proximity and geometry of the cross-talk between neural progenitor cells (NPCs) and endothelial cells (Shen et al., 2004). Similar culture systems have been reported in cancer research (Sontheimer-Phelps et al., 2019). Here, we hypothesized that conventional microfluidic devices could be combined with 3D bioprinting technology to fabricate *ex vivo* tissue mimics with on-chip vascular-like networks.

3D bioprinting technologies are key biomanufacturing methods used to create 3D constructs by sequential deposition of cell-laden bioink layers (Murphy and Atala, 2014; Leberfinger et al., 2019). Several recent examples have demonstrated the promise of 3D bioprinting to create *in vitro* models of human tissues and disease. For example, microextrusion bioprinting was used to generate expansion lattices for neural research (Gu et al., 2018; Lindsay et al., 2019), whereas microextrusion and laser-based bioprinting were used to construct 3D co-culture models of interacting cancer and endothelial cells (Phamduy et al., 2015; Zhou et al., 2016). Despite these exciting advances, the biomaterials commonly used as bioinks, such as alginate and gelatin methacrylate, poorly capture the biochemical complexity and biodegradability of the native ECM.

Previous studies have identified bioink stiffness as a key element for directing cell morphology and differentiation in 3D cultures after bioprinting (Blaeser et al., 2015; Duarte Campos et al., 2015). Cells encapsulated within polymeric 3D microenvironments also require matrix remodeling to spread, migrate, and proliferate. Unfortunately, a trade-off frequently exists between printability and biological outcome when designing bioinks (Duarte Campos et al., 2016). In general, increasing the bioink stiffness can also improve printing precision, whereas cell spreading and differentiation are often improved by decreasing the bioink stiffness. For this reason, proteolytically degradable hydrogels, such as elastin-like protein (ELP) hydrogels, have been successfully engineered to control encapsulated cell phenotype and stemness (Madl et al., 2017). ELP hydrogels are a family of recombinant engineered-protein materials that contain elastin-like repeat units alternating with modular and customizable bioactive domains (Straley and Heilshorn, 2009). The initial stiffness of ELP hydrogels can be tuned by variation of the final concentration of ELP or variation of the crosslinker concentration. For example, in previous work, ELP hydrogel stiffness was varied between 0.5 and 50 kPa in 3–10 wt% ELP hydrogels (Madl et al., 2017). Cell-laden ELP hydrogels were shown to be stable for at least 2 weeks. These materials are proteolytically degradable by collagenases, elastases, and other proteases, resulting in local remodeling of the matrix and enabling cell proliferation over 2 weeks (Chung et al., 2012a; Madl et al., 2017).

**FIGURE 1:**
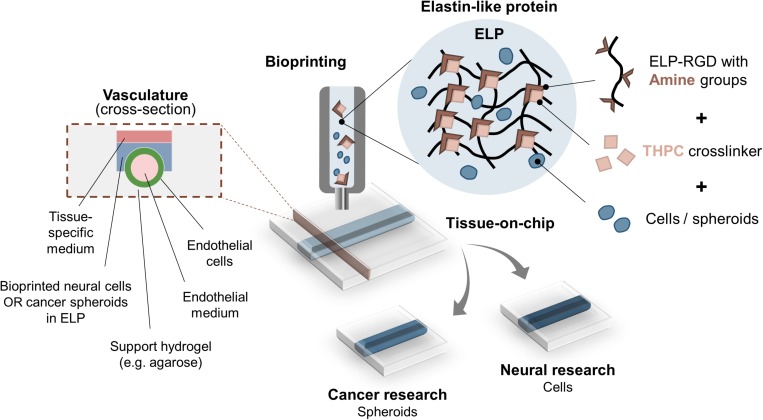
Bioprinting ELP-RGD bioinks with encapsulated cells or spheroids as tissue-on-chip platforms. ELP-RGD bioinks with pendant primary amine groups crosslink in the presence of tetrakis(hydroxymethyl)phosphonium chloride (THPC), which is a tetra-functional, amine-reactive, small molecule crosslinker. Pre-mixed ELP-RGD/THPC bioinks with cells or spheroids are loaded into the bioprinting cartridge and bioprinted directly onto microfluidic chips that enable fabrication of endothelialized vascular-like channels. Two distinct types of culture media are used: endothelial medium is perfused in the lumen of the vascular channel, while neural- or cancer-specific medium is added to the upper culture chamber.

In this study, we explore the feasibility of ELP hydrogels with the fibronectin-derived, cell-adhesive RGD amino acid sequence (ELP-RGD) as bioinks for engineering 3D *in vitro* models with on-chip vascular-like channels (Figure 1). Bioink printability, single-cell and cell-spheroid viability after bioprinting, as well as proof-of-concept bioprinting of a neural tissue-on-chip, were assessed using ELP-RGD hydrogels. Analysis of neural progenitor cell and cancer spheroid survival after bioprinting showed encouraging results after 7 days of culture. Prolonged cultures up to 14 days showed that NPCs spread and cancer spheroids continued growing at a comparable rate as non-bioprinted controls. Preliminary analysis of the endothelialized channels demonstrated distribution of endothelial cells along the entire lumen of the channel.

## Materials and Methods

### Synthesis of ELP-RGD Bioinks and Sacrificial Inks

ELP-RGD hydrogels were synthesized as previously described (Madl et al., 2017; LeSavage et al., 2018). Briefly, ELP was cloned into pET15b plasmids and expressed in BL21(DE3)pLysS *Escherichia coli*. Bacterial cultures containing the plasmids grew to an OD600 of 0.8 before inducing ELP expression with 1 mM isopropyl β-D-1-thiogalactopyranoside (IPTG). After culture, bacteria were harvested, resuspended and lysed by repetitive freeze-thaw cycles. ELP was next purified by inverse temperature cycling, dialyzed, and lyophilized to a solid state. ELP-RGD hydrogels were prepared by first dissolving the lyophilized ELP in phosphate-buffered saline (PBS) to a concentration of 3.75, 5.0, or 6.75 wt%. These ELP solutions were then mixed with a diluted solution of the amine-reactive crosslinker, tetrakis(hydroxymethyl)phosphonium chloride (THPC), in a 4:1 volumetric ratio (ELP solution:THPC solution), resulting in final ELP-RGD hydrogel concentrations of 3, 4, or 5 wt%, respectively. For each hydrogel condition, the THPC solution was initially diluted in PBS such that the hydrogels had a final crosslinking ratio of 0.5:1 (THPC reactive sites:primary amines on ELP). For cell experiments, all components used to prepare the ELP-RGD hydrogels and bioinks were sterile-filtered prior to culture using a 0.22 μm syringe filter.

For bioprinting experiments using the chip design with sacrificial gel-made channels, 5 wt% gelatin was used to encapsulate human umbilical vein endothelial cells (HUVECs) that endothelialized the channel. Stocks of 5 wt% gelatin were prepared by dissolving gelatin powder (Sigma) in deionized water at 37°C, sterile filtering with a 0.22 μm syringe filter, aliquoting in 1 ml tubes, and storing at −20°C. Prior to bioprinting experiments, frozen stocks were thawed at 37°C, mixed with HUVECs, and extruded directly inside the chip with a syringe coupled to an 18G needle. Agarose hydrogels were used to support the formation of gelatin channels. A stock solution of 3 wt% agarose (Bio-Rad) was prepared by dissolving agarose powder in deionized water, followed by autoclave sterilization at 121°C.

For printability experiments using the microextruder, Pluronic F127 (Sigma) was used as a supporting gel in a reservoir. 26 wt% Pluronic gel was prepared by dissolving it in deionized water at 4°C. Molds containing 26 wt% Pluronic were cast prior to ELP-RGD printability testing.

Matrigel was used as non-bioprinted control for encapsulation of cells and spheroids, and for the expansion of cancer spheroids prior to bioprinting experiments. Stocks of Matrigel (#354277, Corning) were stored frozen at −20°C and thawed at 4°C prior to use.

### Bioprinting Setup and Printability of ELP-RGD Bioinks

Printability of ELP-RGD bioinks was tested by drop-on-demand (DoD) and microextrusion bioprinting. DoD experiments were performed using a hand-held bioprinter (DropGun, BlackDrop Biodrucker GmbH, Aachen, Germany), consisting of a hand-held bioink reservoir connected to an air compressor, mounted with a 300 μm micro-valve, and regulated by a controller unit (Duarte Campos et al., 2019). Average drop diameter and weight of ELP-RGD bioinks at concentrations of 3, 4, and 5 wt%, and deionized water (as liquid control) were analyzed. First, the hand-held bioprinter, ELP-RGD hydrogel precursors, and THPC solutions were placed on ice for 15 min prior to the printability experiments in order to slow down the crosslinking speed of ELP-RGD bioinks. ELP-RGD hydrogels at concentrations of 3.75, 5.0, and 6.75 wt% were mixed with a diluted THPC solution in 4:1 volumetric ratio using manual pipetting, and immediately transferred to the bioprinter reservoir. Each printing experiment was performed using a single ejection valve or needle, without use of coaxial needles. These formulations have previously reported to have crosslinking times that vary between 5 and 30 min (Chung et al., 2012b). The loaded hand-held bioprinter was fixed at a 1 cm distance from the printing substrate (glass slide). All materials were dispensed with a valve opening time (gating time) of 450 μs, and at defined pressures of 0.25, 0.5, 0.75, 1.0, or 1.5 bar. Images of the printed drops (*n* = 10 for each material and variable) were recorded immediately after printing, and drop diameter was measured with ImageJ (National Institutes of Health, NIH). For assessing drop weight, 100 drops of each testing material were collected in 500 μl tubes and weighed.

Qualitative printability tests were performed with 3 wt% ELP-RGD bioink by DoD in circular and S shapes (by hand). Additional printability tests by microextrusion were performed using a 3D-bioprinter (Biobot1, Allevi, Philadelphia, PA, United States) mounted with a flat-tip 27G needle. 3 wt% ELP-RGD bioink was pre-mixed with 5 μl green food color, added to one printer head, and extruded in a spiral shape in a Pluronic print bath. Images were taken of both DoD and microextruded specimens.

### Chip Design and Fabrication

Two distinct chips were custom-designed using an online platform (Biofabics Toolbox)^1^, and manufactured (Biofabics, Porto, Portugal): (1) chip with ready-made channel and (2) chip with sacrificial gel-made channel.

**FIGURE 2:**
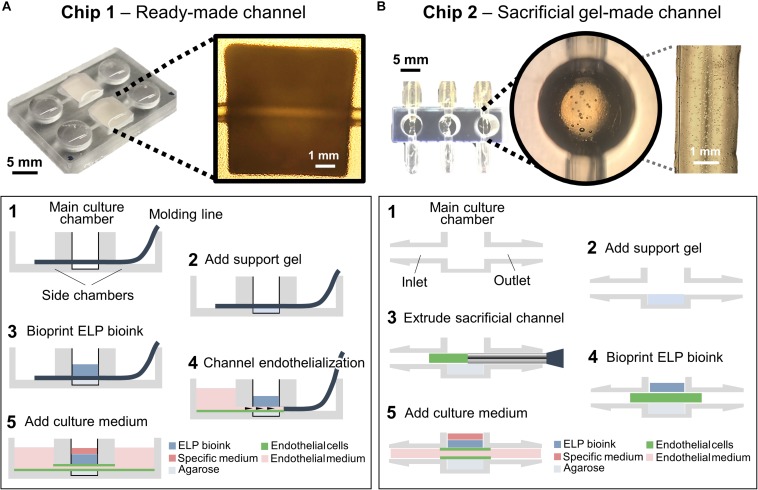
Chip designs used for the biofabrication of printed tissue frameworks with on-chip vascular-like channels. Chips designs with **(A)** ready-made channels, and **(B)** sacrificial gel-made channels were evaluated. Sequence of events (1–5) indicates the necessary working steps to fabricate both types of tissue models. Sacrificial gel-made channels can be used to incorporate endothelial cells that will form monolayers on the channel surface after removing the sacrificial gel. The inset photograph in **(A)** shows the channel formation at step 5. The inset photograph in **(B)** shows the presence of endothelial cells inside the sacrificial gel at step 3.

Chip 1 was designed with tool number 4 of the Biofabics Toolbox (Figure 2A), consisting of a 96-well plate layout with 6.5 mm well diameter, 3.5 mm well height, 11.5 mm channel length, 1 mm channel diameter, 1 mm base thickness, and a square chamber with 6.5 mm width/length. A column of two device units was selected, and all remaining parameters were kept at zero. Each chamber was perfused with a 0.8 mm diameter molding line, which was removed after bioprinting of ELP bioinks.

Chip 2 was designed with tool number 5 of the Biofabics Toolbox (Figure 2B), consisting of a 96-well plate layout with a round chamber with 8 mm chamber height, 5 mm chamber diameter, 2 mm channel diameter, 2 mm channel to chamber bottom distance, and 1 mm base thickness. A column of three device units was selected, and all remaining parameters were kept at zero. The inlet/outlet fittings of each chip were fastened to silicone tubes, which were coupled to syringes as inlets (fresh medium supply, three inlets), or outlets (culture medium waste, three outlets).

### Cell Isolation and Culture

Murine NPCs from micro-dissected dentate gyrus of adult female mice (C57Bl/6) were kindly provided by Prof. Theo Palmer (Stanford Neurosurgery) (Babu et al., 2007). All animal work followed protocols reviewed and approved by the Stanford Administrative Panel on Laboratory Animal Care. NPCs were cultured and expanded in 2D, and encapsulated in 3D ELP-RGD hydrogels for bioprinting experiments, following previously established protocols (Madl et al., 2017; LeSavage et al., 2018). After isolation, NPCs were expanded in maintenance medium [Neurobasal-A (Thermo Fisher Scientific), 2% B27 supplement (Gibco), 2 mM GlutaMAX (Life Technologies), 20 ng/ml fibroblast growth factor 2 (FGF-2), and 20 ng/ml epidermal growth factor (EGF) (PeproTech)] on Poly-L-ornithine and laminin coated tissue culture plastic (Madl et al., 2016). For cell passaging and encapsulation in ELP-RGD bioinks, NPCs were trypsinized, pelleted, resuspended, and counted. For expansion, NPCs were plated at 1 × 10^4^ cells/cm^2^, and, for encapsulation, cells were mixed with hydrogel components to achieve a final density of 1.5 × 10^7^ cells/ml. For bioprinting experiments, NPCs were encapsulated in 3 wt% ELP-RGD bioinks, pre-mixed with THPC, as described above, and loaded into the printing cartridge. After bioprinting, specimens were incubated at room temperature for 15 min, followed by incubation at 37°C for 30 min prior to adding culture medium. Culture medium was replaced every 2 days during cell expansion and every day after bioprinting.

Human induced pluripotent stem cells (hiPSCs, line: 8343.2) were differentiated into cortical neural progenitors following a previously established protocol (Shi et al., 2012). Briefly, hiPSCs were maintained in Essential 8 (E8) medium (Gibco) on Matrigel-coated tissue culture plastic and differentiated in N3 medium consisting of 50% DMEM/F-12, 50% Neurobasal A, 1% N-2 supplement, 2% B-27 supplement, 2 mM GlutaMAX, 1% MEM Non-Essential Amino Acid Solution (NEAA), and 2.5 μg/ml human recombinant insulin (all Thermo Fisher Scientific). N3 medium was further supplemented with 5 μM transforming growth factor beta (TGF-beta) receptor inhibitor [SB-431542 (Tocris)] and 100 nM activin receptorlike kinase 2 (ALK2) and ALK3 inhibitor [LDN-193189 (Stemgent)] for the first 11 days of culture. At day 12, hiPSCs were dissociated with Cell Dissociation Solution (Sigma) and plated on pre-coated plates with 50 μg/ml Poly-D-Lysine (Sigma) and 5 μg/ml Laminin (Roche). Next, hiPSC-derived NPCs were further cultured in N3 medium without SB-431542 or LDN-193189 until day 16. Prior to bioprinting experiments, hiPSC-NPCs were dissociated and encapsulated in 3 wt% ELP-RGD bioinks pre-mixed with THPC at a density of 1.5 × 10^7^ cells/ml. After bioprinting, specimens were incubated at room temperature for 15 min, followed by incubation at 37°C for 30 min prior to adding culture medium. Culture medium was replaced daily before and after bioprinting.

HUVECs were purchased from PromoCell. HUVECs were expanded in EGM-2 growth medium (Lonza) containing 0.04% hydrocortisone, 0.4% human FGF-2, 0.1% vascular endothelial growth factor (VEGF), 0.1% recombinant analog of insulin-like growth factor (R3-IGF-1), 0.1% ascorbic acid, 0.1% human EGF, 0.1% gentamicin sulfate-amphotericin (GA-1000), 0.1% heparin, and 2% fetal bovine serum (FBS). For cell passaging and encapsulation in gelatin, HUVECs were trypsinized, pelleted, resuspended, and counted. HUVECs were plated at 1 × 10^4^ cells/cm^2^ for expansion. For encapsulation, cells were mixed with 5 wt% gelatin to achieve a final density of 1 × 10^7^ cells/ml. Culture medium was replaced every 2 days during cell expansion and every day after bioprinting.

Human premalignant breast epithelial cells (MCF10ATs) were kindly provided by Jan Liphardt (Stanford Bioengineering) and expanded, following a previously published protocol (Leung and Brugge, 2012). Briefly, MCF10ATs were expanded in DMEM/F-12 medium (Gibco) supplemented with 100 μg/ml EGF (Gibco), 1 mg/ml hydrocortisone (Sigma), 1 mg/ml cholera toxin (Sigma), 10 mg/ml insulin (Sigma), and 100x penicillin/streptomycin (Gibco). For cell passaging and encapsulation in Matrigel, MCF10ATs were trypsinized, pelleted, resuspended, and counted. For expansion, MCF10ATs were plated at 1 × 10^4^ cells/cm^2^, and for encapsulation, cells were mixed with Matrigel to achieve a final density of 5 × 10^5^ cells/ml. After 7 days culture in Matrigel, MCF10AT spheroids with approximately 50 μm diameter, containing dozens of cells, were harvested for bioprinting experiments. Briefly, MCF10AT-laden Matrigel cultures were incubated in 5 mM EDTA in PBS for 45 min on ice. Dissociated Matrigel, containing MCF10AT spheroids, was pelleted and resuspended in basal medium. MCF10AT spheroids were encapsulated at a density of 1 × 10^7^ spheroids/ml in 3 wt% ELP-RGD bioinks pre-mixed with THPC, as described above, and loaded into the printing cartridge. After bioprinting, specimens were incubated at room temperature for 15 min, followed by incubation at 37°C for 30 min prior to adding culture medium. Culture medium was replaced every 2 days during expansion and daily after bioprinting.

### Bioprinting of Cells and Spheroids in Tissue-on-Chips

Before bioprinting experiments, chips were washed thrice in 70% ethanol and sterile PBS. Cell and spheroid-laden 3 wt% ELP-RGD bioinks were prepared as described above and bioprinted by DoD. For chip 1, bioprinting of bioinks was performed prior to channel endothelialization. First, each main culture chamber was filled with 100 μl bioink. After crosslinking, HUVECs suspended in culture medium were added to one of the supporting chambers, and the molding line was carefully removed, allowing the HUVEC suspension to fill the channel by capillary action. Subsequently, chips were flipped and incubated bottom-up at 37°C for 4 h to allow for cell seeding on the upper segment of the channel, followed by upright incubation at 37°C for 5 days. Culture media for both cell types was replaced daily.

Bioprinting into chip 2 was performed after channel formation. First, sterile 18G needles were inserted in the chips through the side tube connectors. Twenty microliter 3 wt% agarose was cast in the bottom of each main culture chamber to support the formation of a HUVEC-laden gelatin channel. HUVEC-laden 5 wt% gelatin was prepared as described above and extruded via syringe inside the culture chamber at room temperature. After gelation of the gelatin channel, 50 μl cell-laden ELP-RGD bioinks were bioprinted by DoD into each chamber and allowed to crosslink for 15 min at room temperature. Next, chips were flipped and incubated bottom up at 37°C for 4 h. At this step, gelatin channels melted and HUVECs sedimented by gravity on the upper segment of the channel. Chips were once again flipped and incubated upright for 5 days. Syringes with fresh EGM-2 medium for HUVEC culture were connected to one side of the chamber, and empty syringes were connected to the opposite side to collect media waste. Cell-specific culture medium was added to the upper part of the main chamber, and both culture media were replaced daily.

### Viability Staining

Three, seven, and fourteen days after cell and spheroid encapsulation in hydrogels and bioprinting experiments, specimens were removed from the incubator, washed once with PBS, and stained with Live/Dead viability staining (Thermo Fisher Scientific). Staining conditions were optimized for cells encapsulated in Matrigel and ELP-RGD. For Matrigel samples, stock solutions were diluted to 0.5 μg/ml calcein and 2 μl/ml ethidium homodimer-1 (EthD-1) in PBS, and incubated for 30 min at 37°C. Cells in 3 wt% ELP-RGD were incubated in solutions of 1 μl/ml calcein and 2 μl/ml EthD-1 diluted in PBS for 30 min at 37°C. After incubation, samples were washed once with PBS and imaged using a Leica SPE confocal microscope. Percent (%) viability was calculated based on the number of counted live and dead cells (Equation 1). Three independent biological replicates were used for each time point and variable (*N* = 3).

Percent (%) viability of cells and spheroids.

(1) $$\%Viability=\frac{L\,i\,v\,e\,C\,e\,l\,l\,s}{L\,i\,v\,e\,C\,e\,l\,l\,s+D\,e\,a\,d\,C\,e\,l\,l\,s}\times 100$$

### Immunocytochemical Staining

For immunocytochemistry, samples were fixed with 4% paraformaldehyde in PBS for 30 min at 37°C. Samples were permeabilized with 0.25% Triton X-100 in PBS (PBST) for 1 h at room temperature. The samples were then blocked with 5% bovine serum albumin (BSA) and 5% goat serum in PBST for 3 h at room temperature. Primary antibodies against sex determining region Y-box 2 (Sox2) (1:400, rabbit, Cell Signaling Technology, 23064S) and/or cluster of differentiation 31 (CD31) (1:200, mouse, PECAM-1, Sigma, P8590) were diluted in PBST containing 2.5% goat serum, added to samples, and incubated overnight at 4°C. The samples were then washed thrice with PBST, incubated with secondary antibodies [AlexaFluor488 (goat anti-rabbit, 1:500); AlexaFluor647 (goat anti-mouse, 1:500); and/or tetramethylrhodamine (TRITC) phalloidin 532 (F-actin, 1:500)], and counterstained with 4′,6-diamidino-2-phenylindole dihydrochloride (DAPI) overnight at 4°C. Samples were washed thrice with PBST and imaged using a Leica SPE confocal microscope. Three independent biological replicates were used for each time point and variable (*N* = 3).

### Analysis of Confocal Images

Confocal images were analyzed with ImageJ (NIH). Images recorded with the same magnification (20× objective) were transformed into 8-bit images. Color channels of each image were split, and the threshold was manually adjusted prior to measuring percent cell area values. Total cell area was calculated as a function of the total image area of 2.9 × 10^5^ μm^2^. Three independent biological replicates were used for each time point and variable (*N* = 3).

### Statistical Analysis

Comparisons between two experimental groups were made using two-tailed Student’s *t*-tests (Prism 8, version 8.3.0, GraphPad Software). Comparisons between three or more experimental groups were made using one-way ANOVA with Tukey’s *post hoc* test. Statistical significance was considered as ^∗^*p* < 0.05, ^∗∗^*p* < 0.005, and ^∗∗∗^*p* < 0.001. Independent biological replicates and exact *P*-values are indicated in each Figure including statistical analyses. Experimental data are presented as means ± standard deviation.

## Results

### ELP-RGD Printability by Drop-on-Demand and Microextrusion Bioprinting

**FIGURE 3:**
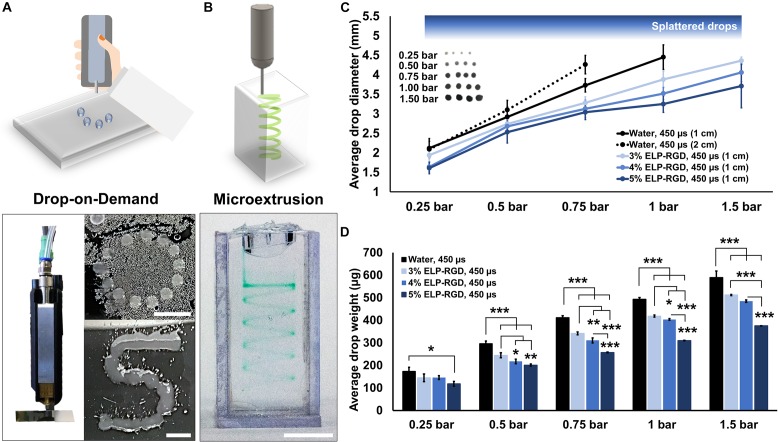
Printability of ELP-RGD bioinks. **(A)** Qualitative drop-on-demand (DoD) printability tests of 3 wt% ELP-RGD printed as single drops into circular and S shapes. Scale bars represent 5 mm. **(B)** Qualitative microextrusion printability test of 3 wt% ELP-RGD in a spiral shape within a Pluronic bath. Scale bar represents 5 mm. **(C)** Average single drop diameter of deionized water, 3, 4, and 5 wt% ELP-RGD printed by DoD at applied pressures ranging from 0.25 to 1.5 bar (*n* = 10). **(D)** Average single drop mass of deionized water, 3, 4, and 5 wt% ELP-RGD printed by DoD at applied pressures ranging from 0.25 to 1.5 bar (*n* = 3, 100 drops each). Valve opening time (gating time) was fixed at 450 μs for all experiments. Statistical significance marked as *p < 0.05, **p < 0.005, and ***p < 0.001 (One-way ANOVA with Tukey’s *post hoc* test).

The printability of ELP-RGD bioinks at varying concentrations was tested by DoD (Figure 3A) and microextrusion bioprinting (Figure 3B). For all tests, the ELP-RGD engineered protein was solubilized in PBS and pre-mixed with the tetra-functional crosslinker THPC at a 0.5:1 stoichiometric ratio between THPC functional groups and primary amines in the protein. DoD printability tests were performed with a hand-held bioprinter connected to an air compressor, which allowed for hand-guided dispensing of single bioink drops in circular and S shapes. Microextrusion printability tests were performed using a 3D-bioprinter, which allowed for continuous extrusion of the bioink in spiral shapes submerged in a Pluronic print-bath. The smallest diameter observed for single drops of ELP-RGD bioinks printed by DoD was 1.4 mm (5 wt% ELP-RGD at 0.25 bar, Figure 3C). By increasing the applied pressure during printing, it was possible to increase the drop size without varying bioink concentration or nozzle diameter. Drops larger than 4 mm in diameter were difficult to measure due to impaired droplet integrity wherein large drops tended to splatter into smaller drops. Nevertheless, drop size and weight continued to increase upon increasing the applied pressure, as shown in Figures 2C, 3D. Interestingly, the mass of the printed drops decreased with increasing ELP-RGD concentration and constant pressure. For example, at 0.75 bar, the 3 wt% ELP-RGD formed single drops with an average mass of 342 μg, whereas that of 4 wt% and 5 wt% ELP-RGD inks had average single drops of 311 and 257 μg, respectively.

### Chip Design and Visualization of Perfused Channels

Toward the long-term goal of culturing bioprinted 3D tissue models with vascular-like channels, we evaluated two different chip designs and fabrication protocols. In one method, a simple, easy-to-use chip design with ready-made channels was evaluated (Figure 2A). This chip comprised two main culture chambers, where the tissue models were bioprinted, and four supporting chambers, where cell culture medium was added. Two plastic molding lines with 0.8 mm diameters were inserted in each chip by traversing the main culture chambers and the two supporting chambers connected to them. After DoD bioprinting of the ELP-RGD layers onto the plastic wire, HUVECs suspended in medium were added to one of the sides of the chip and were subsequently seeded in the inner wall of the channel by removing the plastic line. As a second method, a chip design comprising culture chambers with tubing connectors suitable for dynamic perfusion culture was evaluated (Figure 2B). This type of chip design allowed for HUVEC seeding in the vascular-like channel within a sacrificial gelatin hydrogel. The ELP-RGD ink was bioprinted directly on top of the sacrificial gelatin to form an upper layer of printed tissue. After the last bioprinting step, the chips were placed in an incubator at 37°C allowing for further crosslinking and solidification of the ELP-RGD bioink, and simultaneous fluidization and removal of the sacrificial gelatin channel.

### Cell and Spheroid Viability in ELP-RGD Bioinks

**FIGURE 4:**
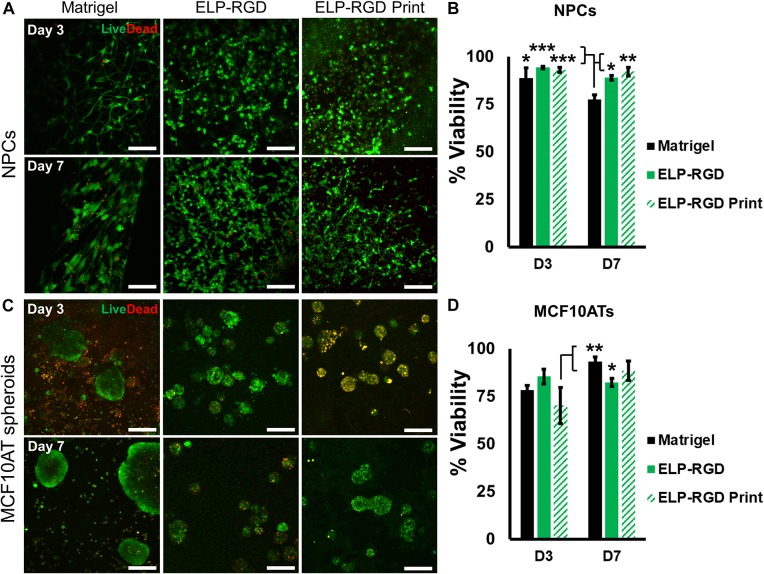
Viability of dissociated cells and spheroids with and without bioprinting. Live cells are stained with calcein (green) and dead cells are stained with ethidium homodimer-1 (red). **(A)** Live/Dead staining of murine neural progenitor cells (NPCs) on days 3 and 7 within Matrigel (not bioprinted), 3 wt% ELP-RGD (not bioprinted), and 3 wt% ELP-RGD after DoD bioprinting. Scale bars represent 100 μm. **(B)** Quantified viability of NPCs at days 3 and 7. Statistical significance marked as *p < 0.05, **p < 0.005, and ***p < 0.001 (*N* = 3, One-way ANOVA with Tukey’s *post hoc* test). **(C)** Live/Dead staining of human premalignant breast epithelial cell spheroids (MCF10ATs) in Matrigel (not bioprinted), 3 wt% ELP-RGD (not bioprinted), and 3 wt% ELP-RGD after DoD bioprinting at days 3 and 7. Scale bars represent 100 μm. **(D)** Quantified viability of MCF10ATs at days 3 and 7. Statistical significance marked as *p < 0.05, and **p < 0.005 (*N* = 3, One-way ANOVA with Tukey’s *post hoc* test).

Several variables have been reported to impact cell viability during different bioprinting methods, including bioink viscosity, printing nozzle size, and applied printing pressure (Duarte Campos et al., 2015; Dubbin et al., 2017). Therefore, an important step toward developing a bioprinted *in vitro* tissue-on-chip model is to evaluate cell viability. In this study, cell and spheroid viability was analyzed after DoD bioprinting using dissociated NPCs (Figure 4A) and MCF10AT spheroids (Figure 4C). Cells and spheroids encapsulated in 3 wt% ELP-RGD bioinks and Matrigel without bioprinting were included as controls. NPC survival in 3 wt% ELP-RGD was greater than 88.9% in both experimental groups, including non-bioprinted and bioprinted samples (Figure 4B). The lowest NPC survival of about 77.4% was observed in non-bioprinted Matrigel controls after 7 days of culture. The overall MCF10AT spheroid viability rates at day three of culture were lower compared to NPC cultures (Figure 4D). The lowest MCF10AT spheroid survival rate observed was about 70.2% 3 days after bioprinting. Interestingly, upon further time in culture, bioprinted MCF10AT viability rates increased to about 88.3% at day seven of culture. This cell viability at 7-days post-printing was not statistically significantly different than the 7-day viability without printing in either ELP-RGD (82.3%) or Matrigel (93.3%).

### Endothelialization of the Channels Contained in the Culture Chips

**FIGURE 5:**
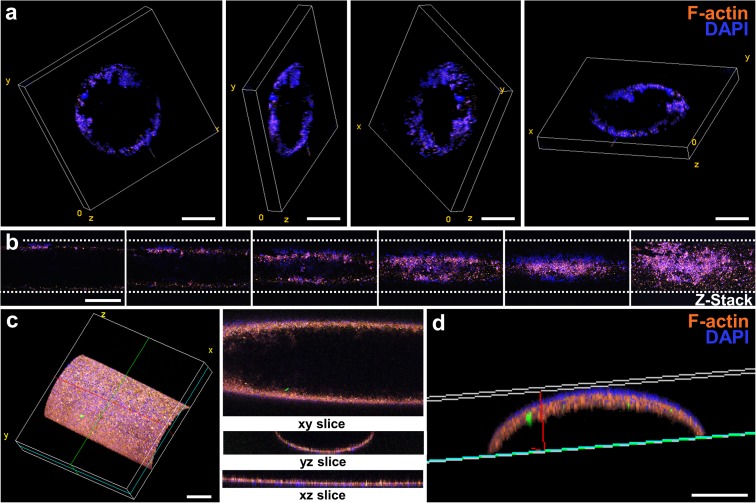
Endothelialization of the luminal wall of the chip. **(a)** Sequence of cross-sectional confocal images showing the position of HUVECs seeded within the channel. Scale bars represent 250 μm. **(b)** Sequence of confocal images taken lengthwise to the channel. The image at the far-right shows a z-stack projection of the sequence of images at varying z heights. Scale bar represents 500 μm. **(c)** 3D projection of an endothelialized channel showing the cross-section views at the xy, yz, and xz planes. Scale bar represents 250 μm. **(d)** Cross-section yz view of **(d)**. Scale bar represents 100 μm.

Perfused and endothelialized tissue-on-chips are important components of many *ex vivo* tissue models. In neural research, such models may be used to mimic the neural stem cell niche to study the requirements for stem cell maintenance and activation, or in the future to recapitulate the blood-brain-barrier to study the penetration of new drugs across the barrier. In cancer research, these models may be used to investigate cancer cell migration and metastasis through blood vessels. In this work, we evaluated the endothelialization of chips with a ready-made channel design. After HUVEC seeding, culture, and fixation, entire chips were stained, sectioned, and imaged by confocal microscopy. Cross-section views with different 3D perspectives of endothelialized channels are shown in Figure 5a. A HUVEC monolayer was formed along the inner wall of the channel contained in the culture chip. Next, the endothelialized channel was imaged lengthwise to show the presence of HUVECs at different z heights (Figure 5b) and in 3D perspective (Figure 5c). A compiled view of the y-z cross-section of the channel (Figure 5d) demonstrates a uniform distribution of HUVECs along the inner wall of the channel.

### Morphology of Printed NPCs and Premalignant Breast Epithelial Spheroids

**FIGURE 6:**
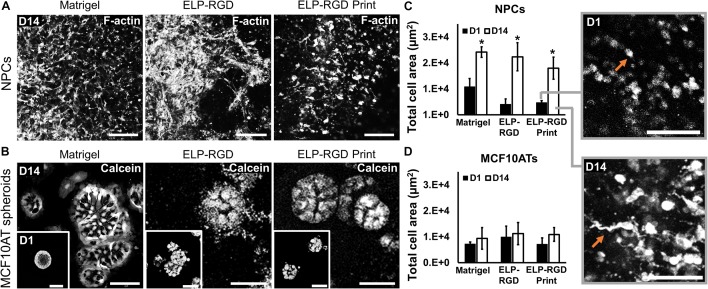
Morphology of dissociated NPCs and MCF10AT spheroids after 14 days of culture within 3D hydrogels. **(A)** Confocal images of NPCs cultured for 14 days in Matrigel, 3 wt% ELP-RGD without bioprinting, and 3 wt% ELP-RGD after DoD bioprinting. Scale bars represent 100 μm. **(B)** Confocal images of MCF10AT spheroids cultured for 14 days in Matrigel, 3 wt% ELP-RGD without bioprinting, and 3 wt% ELP-RGD after DoD bioprinting. Scale bars represent 50 μm. Insets show MCF10AT spheroids at day 1 of culture in the respective material. Scale bars represent 100 μm. **(C)** Total cell area of stained NPCs in confocal images taken at days 1 and 14 of culture. Statistical significance between days 1 and 14 of each material marked as *p < 0.05 (*N* = 3, two-tailed *t*-test, unpaired). Insets are magnified views of NPCs cultured in 3 wt% ELP-RGD after bioprinting, showing round cells at day 1 and spread cells at day 14 (orange arrows). Scale bars represent 100 μm. **(D)** Total cell area of stained MCF10AT spheroids in confocal images taken at days 1 and 14 of culture. One-way ANOVA with Tukey’s *post hoc* test did not show statistical significance between groups.

To develop tissue-on-chips suitable for use as *ex vivo* tissue models, it is important to evaluate cell and spheroid morphological changes during culture. NPCs encapsulated in bioprinted and non-bioprinted matrices showed morphological changes from rounded at day 1 to elongated at day 14 (Figure 6A). In contrast, MCF10AT spheroids did not show morphological alterations throughout the culture period (Figure 6B). F-actin staining of NPCs encapsulated in each matrix, both with or without bioprinting, showed a significant increase (^∗^*p* < 0.05) in total cell area from days 1 to 14, suggesting increased cell spreading and elongation (Figure 6C). No statistically significant differences in NPC cell-spreading were observed between the various matrices, with or without bioprinting. Similarly, MCF10AT spheroid size was not significantly different within the different matrices. In addition, total cell area of MCF10AT spheroids encapsulated in bioprinted and non-bioprinted matrices did not significantly change between days 1 and 14 of culture (Figure 6D).

### Bioprinted on-Chip Co-culture With Vascular-Like Channel

**FIGURE 7:**
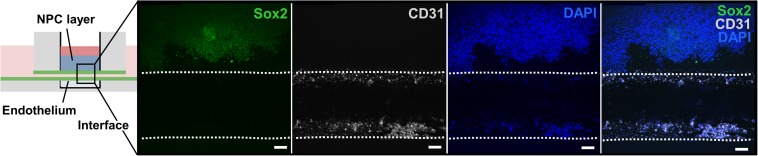
Bioprinted tissue-on-chip with vascular-like channel. Schematic of the tissue-on-chip design. Confocal images of hiPSC-NPCs encapsulated within 3 wt% ELP-RGD bioprinted on top of a channel seeded with HUVECs and cultured for 5 days. hiPSC-NPCs were visualized with Sox2 immunostaining, HUVECs were visualized with CD31 immunostaining, and all cells were visualized by nuclear staining with DAPI. Scale bars represent 50 μm.

After assessing bioink printability, cell viability after bioprinting, and cell morphological changes during *in vitro* culture, we developed and analyzed a proof-of-concept *ex vivo* model of the neural stem cell niche that included both HUVECs and hiPSC-derived NPCs. For this experiment, the chip with the ready-made channel was used. hiPSC-NPCs were encapsulated in 3 wt% ELP-RGD bioinks and bioprinted by DoD over the main chamber of the chip. After bioprinting of hiPSC-NPCs, HUVECs suspended in medium were added to one side chamber of the chip, and the perfusion line was removed to induce HUVEC seeding within the vascular-like channel. Confocal images of the tissue chips after 5 days of culture showed the presence of hiPSC-NPCs (Sox-2-positive, a marker of neural progenitor cell pluripotency) within the printed ELP-RGD hydrogel and the presence of HUVECs (CD31-positive, also known as platelet endothelial cell adhesion molecule (PECAM-1), a common endothelial marker) within the vascular-like channel (Figure 7).

## Discussion

The goal of this study was to explore the feasibility of using ELP-RGD hydrogels as bioinks to fabricate 3D tissue models on microfluidic chips with vascular-like channels. Following a previously established protocol (LeSavage et al., 2018), we synthesized ELP-RGD hydrogels and tested their printability as bioinks. First, the printability by DoD was tested using a hand-held bioprinting device. DoD bioprinting was chosen for this study, because it has been previously reported as a more cell-friendly and freeform method compared to, for example, microextrusion bioprinting (Blaeser et al., 2017). Drop size and weight strongly varied depending on the concentration of ELP-RGD bioink (3, 4, or 5 wt%), applied printing pressure, and distance to the printing substrate. This outcome is in accordance with previous studies, which used other hydrogels with similar rheological properties, such as polysaccharides (Blaeser et al., 2015; Duarte Campos et al., 2019). Comparable ELP-based bioinks were tested in previous works using microextrusion bioprinting (Salinas-Fernández et al., 2019). In this previous work, the geometric printability outcomes highly depended on the concentration and amino acid sequence of the ELP bioink. In our work, qualitative printability experiments by microextrusion showed well-defined 3D shapes with complex and freeform geometry (spirals, Figure 2B).

Survival of neural cells and cancer spheroids encapsulated in 3 wt% ELP-RGD was assessed after DoD bioprinting. We found that the average neural cell survival (88.9%) was in accordance with other studies that used the same bioprinting method in combination with other bioinks and cell types (Forget et al., 2017; Kreimendahl et al., 2017; Ng et al., 2017). Contrastingly, average cancer spheroid survival after bioprinting (70.2%) was markedly lower compared to neural cell survival. This result may be due to the larger diameter of cellular MCF10AT spheroids (approximately 50 μm) compared to dissociated, single NPCs (about 10 μm). It is well-known that increased fluid stresses within the printing nozzle can negatively affect cell viability (Aguado et al., 2012; Blaeser et al., 2015; Foster et al., 2017; Lindsay et al., 2019). Nozzle shape, orifice diameter, bioink viscosity, cell density in the bioink, and cell size are parameters that will influence fluid stress, thus making cell aggregates more susceptible to damage (Cidonio et al., 2019). Despite this, cell aggregates with large diameters, including cell clusters like spheroids, may be helpful for the fabrication of *ex vivo* tissue models (Li and Kumacheva, 2018). Therefore, future work should further evaluate the possible correlation between cell aggregate diameter and low viability post printing. Despite this decrease in cell viability immediately post-printing, by day 7 the printed cultures had recovered and displayed viability and sizes similar to those that had not been exposed to printing. Importantly, day 7 viability and day 14 spheroid size were statistically similar for cultures grown in Matrigel or bioprinted in ELP-RGD. Together, these data suggest that this engineered bioink hydrogel can support the long-term culture of these cancer spheroids after direct bioprinting on a microfluidic chip.

NPCs are a promising cell source for recreating a model of the neural environment. The native neural stem cell niche includes a vascular network that is critical in maintenance of NPC stemness (Tavazoie et al., 2008), with known cross-talk between endothelial cells and NPCs (Schänzer et al., 2006). *In vitro* differentiation toward astrocytes and neurons can be challenging in 3D, and thus, bioinks and the bioprinting process should ideally not restrict NPC differentiation potential. An important aspect of a bioink to support NPC differentiation is to enable cell-cell contact by cell-mediated matrix remodeling (Madl et al., 2019). In this work, we showed that NPCs elongated to spindle-like shapes in 3 wt% ELP-RGD bioinks after bioprinting and kept growing up to 14 days in culture. This morphology suggests that the encapsulated NPCs are able to remodel the printed matrix and make cell-cell contacts that are required for maintenance of stemness. These results are further confirmed by the continued expression of the neural stem cell marker Sox2. The use of ELP-RGD hydrogels as bioinks is an advancement in the fabrication of on-chip tissues to mimic aspects of the neural-vascular interface. Many of these previous studies have used hydrogels that allow little to no cell-driven matrix remodeling, such as agarose (Gasperini et al., 2014; Duarte Campos et al., 2016; Gu et al., 2018).

Vascularization is a frequent goal for the fabrication of engineered tissues *in vitro* (Zhang et al., 2016). For this reason, we used custom-designed on-chip platforms that contained vascular-like channels and that allowed for integrated 3D bioprinting. Microfluidic platforms have been used in previous studies, for example, to investigate cancer metastasis *in vitro* (Sontheimer-Phelps et al., 2019). However, these technologies are difficult to combine with current 3D bioprinting strategies, given that chamber and channel size is in the micrometer range. Therefore, we designed on-chip platforms consisting of culture chambers and channels in the millimeter range, which can be easily accessed with a bioprinter. Two distinct chip designs were considered for their suitability with tissue bioprinting. The first design, which was used for static culture, was simpler and easier to use, as it required fewer preparation steps. HUVECs were successfully seeded onto the inner walls of the channels in these platforms. We showed that HUVECs were distributed along the entire wall of these channels. Microvascular *in vitro* models containing several cell types and layers that resemble native (micro)vessels were previously reported (Jaeger et al., 2013; Cochrane et al., 2019). Although *in vitro* vascular-like structures are typically generated using only endothelial cells, such as HUVECs, more complex 3D models will require triple-layered vascular structures, including fibroblasts, endothelial, and smooth muscle cells (Gao et al., 2019). A limitation of our study was the use of only endothelial cells to cover the inner wall of the channels. Future experiments will consider improving the *in vivo* relevance of the channels by inducing additional cell types. Additionally, functional characteristics of the endothelialized layer, including permeability to nutrients, need to be assessed (Bang et al., 2017).

Finally, as proof-of-concept, a tissue-on-chip was fabricated by bioprinting hiPSC-derived NPCs using protein-engineered-bioinks onto a device with a vascular-like, endothelialized channel. Research studying the cross-talk between endothelial cells and NPCs uses random co-culture mixtures or transwell inserts (Shen et al., 2004), but these do not properly recapitulate the spatial proximity and geometry of these cells in the native stem cell niche, thus resulting in non-physiological concentration gradients of secreted, diffusible factors and inappropriate presentation of cell-surface receptors. In this study, we suggest that a combination of bioprinting and on-chip technologies can be used to realize NPC/HUVEC co-cultures with physiologically relevant geometric patterning. In our previous work, we found that NPCs would lose their Sox2 expression within 2 days in matrices that could not support stemness maintenance (Madl et al., 2017). In this experiment, we showed that both cell types remained viable over 5 days of culture, retained their proper positioning within the co-culture device, and maintained NPC stemness. Although the bioprinter used in this study is hand-held, our results provide a proof-of-concept demonstration that (i) it is feasible to bioprint ELP-RGD bioinks with precision and spatial control, (ii) these bioinks are compatible with the materials commonly used to fabricate microfluidic devices, and (iii) these bioinks are compatible with several cell types of interest. Importantly, this bioprinting technology can be easily mounted onto a stereotactic controller (Blaeser et al., 2015; Duarte Campos et al., 2019). Such automated dispensing of cell-laden hydrogels within microfluidic devices would offer higher spatial precision, greater reproducibility, and more efficient scale-up compared to manual pipetting.

Here we have successfully demonstrated that bioprinted NPCs are able to maintain their stem-like phenotype and hence remain Sox2-positive during co-culture with endothelial cells within a microfluidic device. This platform is well-suited for future cell biology studies investigating the cross-talk between ECs and NPCs that mediate stem cell maintenance and activation.

## Conclusion

In this work, we used protein-engineered ELP-RGD hydrogels as bioinks for producing *in vitro* tissue-on-chip platforms. We showed that ELP-RGD bioinks are dispensable by DoD and microextrusion, and that 3D constructs can be generated. Assessment of neural progenitor cell and cancer spheroid survival after bioprinting showed encouraging results after 7 days of culture. Prolonged cultures up to 14 days showed that NPCs spread, and cancer spheroids continue growing at a comparable rate as non-bioprinted controls. Preliminary analysis of the endothelialized channels demonstrated distribution of endothelial cells along the entire lumen of the channel. The results presented here represent a first step in combining ELP engineered hydrogels with 3D bioprinting technologies and on-chip platforms for establishing vascularized *in vitro* tissue models. In the future, these platforms may be further developed for *in vitro* studies of interactions between the vascular interface and patterned, three-dimensional tissue mimics.

## Data Availability Statement

The datasets generated for this study are available on request to the corresponding author.

## Author Contributions

DD and SH: conceptualization. DD, CL, JR, BL, AS, and BK: data acquisition. DC: writing-original draft preparation. DD, CL, JR, BL, AS, BK, RR, PC, AB, and SH: writing and editing and final approval of manuscript. DD, SH, PC, and RR: funding acquisition.

## Conflict of Interest

PC and RR were employed by the company Biofabics Lda. PC was also shareholder of the company Biofabics Lda. The remaining authors declare that the research was conducted in the absence of any commercial or financial relationships that could be construed as a potential conflict of interest.

## Footnotes

1 www.biofabics-toolbox.com
